# ACE Inhibitor-Induced Angioedema following Cervical Spine Surgery

**DOI:** 10.1155/2017/4268962

**Published:** 2017-03-01

**Authors:** Faris Hannoodi, Hussam Sabbagh

**Affiliations:** Wayne State University, Detroit, MI, USA

## Abstract

Angioedema is a well-known side effect of angiotensin converting enzyme inhibitors (ACEi). However, ACE inhibitors induced angioedema after cervical surgery is a rare condition. They result in increased levels of circulating bradykinins. Rare cases of angioedema following local trauma in patients using ACE inhibitors have been published. We present such a case. A 54-year-old Caucasian female with a history significant for hypertension, controlled with lisinopril, was admitted for routine cervical spine surgery. She has severe degenerative cervical disc disease and was admitted to the hospital for an elective cervical diskectomy. The patient failed weaning off the ventilator on multiple attempts postoperatively. There were no observed symptoms of an allergic reaction. A CT scan of the neck showed extensive soft tissue edema at the level of the arytenoids. Dexamethasone was given to reduce the edema without successful resolution. On review of her medications, it was found that the patient was resumed on lisinopril following the procedure. It was subsequently discontinued. By the following day the patient had a positive leak around the ET tube cuff and patient was successfully extubated.

## 1. Introduction

Angioedema is a well-known side effect of angiotensin converting enzyme inhibitors (ACEi). Angioedema occurs because ACE inhibitors impair bradykinin degradation, leading to increase in bradykinin levels. Bradykinin in turn leads to increased vasodilation and vascular permeability, resulting in angioedema. Mast cells are not involved in this pathway. As a result, histamine is not produced, therefore symptoms of pruritus and urticaria do not present.

ACE inhibitor-induced angioedema tends to involve the periorbital region and structures within the oral cavity, oropharynx, and larynx [1, 2]. ACE inhibitor-induced angioedema after cervical surgery is a rare condition. A few cases of angioedema following local trauma in patients using ACE inhibitors have been published [3–7]. We present an interesting case of severe angioedema causing airway obstruction after anterior cervical surgery in a patient using ACE inhibitors.

## 2. Case Report

A 54-year-old Caucasian female with a medical history significant for hypertension, hyperlipidemia, cervical disc disease, and depression was admitted for routine cervical spine surgery. She has never smoked and does not drink alcohol. Her medications included lisinopril 10 mg, atorvastatin 40 mg, citalopram 20 mg, and furosemide 20 mg. She has severe degenerative disk disease at C4–C7, with herniated nucleus pulposus. The patient was admitted to hospital for an elective cervical disk arthroplasty with diskectomy at C4 to C7 and fusion at C5–C7.

**Figure 1:**
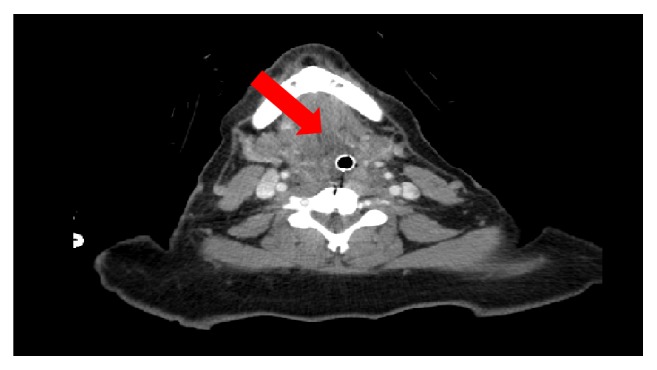
Soft tissue edema demonstrated around the endotracheal tube.

The patient failed weaning off the ventilator on multiple attempts postoperatively. There were no observed symptoms of an allergic reaction. Her vital signs and laboratory tests were unremarkable. CT scan of the neck showed extensive edema at the level of the arytenoids, but no retropharyngeal hematoma or abscess were noted (Figure 1). A bronchoscopy confirmed arytenoid edema.

Dexamethasone 8 mg was given every 6 hours for 4 days to reduce the edema without successful resolution. On review of her medications, it was found that the patient was on lisinopril following the procedure for the treatment of hypertension. It was subsequently discontinued. The following day, the patient was weaned and successfully extubated.

## 3. Discussion

In our case the surgery most likely resulted in marked bradykinin release in a patient who was already on an ACE inhibitor. The release of bradykinin, in addition to decreased bradykinin catabolism as a result of ACE inhibitor therapy, has precipitated angioedema. The definitive treatment of angioedema is to completely stop the offending medication, in this case lisinopril.

There are several risk factors that can contribute to ACE inhibitor-induced angioedema, including previous angioedema, age above 65, NSAID use, female sex, smoking, seasonal allergies, certain immunosuppressants (sirolimus and everolimus), underlying C1 inhibitor deficiency or dysfunction, history of ACE inhibitor-induced cough, and surgery [8–10]. The relevant risk factors to our case are female sex and surgery, though no further testing was carried out to look for C1 inhibitor deficiency.

The areas affected by ACE inhibitor-induced angioedema are the face, mouth, upper airway, and intestine. In the reported cases where angioedema occurred following surgical procedures, the affected areas involved the oral cavity and upper airway [5–7]. This is likely due to local trauma as a result of the cervical spinal surgery. This is consistent with reviewed literature since head and neck surgery appear to increase the incidence of ACE inhibitor-induced angioedema to the oropharynx and upper airway. Of the three surgical cases reported, two required definitive airways to be present to prevent airway compromise, one of which failed intubation and required a tracheotomy [5, 7]. Only one reported case did not require intubation to secure the airway [6].

The mainstay of management is to secure the airway, discontinue the ACE inhibitor, and give systemic steroids. Other additions can include giving epinephrine and antihistaminics [5]. Persistent symptoms may require synthetic bradykinin B2-receptor antagonist. In the reviewed cases, however, the management was similar to that of our case. The airway was secured and systemic steroids were given. In one case, Benadryl and epinephrine were administered as angioedema was treated as an allergic reaction. In another case systemic steroids were given for a period of 7 days for complete resolution [5–7].

## 4. Conclusion

To sum up, although ACE inhibitor-induced angioedema is rare in the surgical setting, the complications can be life-threatening. ACE inhibitors should be discontinued in ALL patients undergoing neck surgery, regardless of the presence of specific risk factors. There is no sense in taking any risks.
